# Influence of SARS-CoV-2 Virus Infection on the Course of Psoriasis during Treatment with Biological Drugs

**DOI:** 10.3390/medicina57090881

**Published:** 2021-08-27

**Authors:** Magdalena Mroz, Szymon Mućka, Martyna Miodońska, Dominika Ziolkowska, Ewa Hadas, Andrzej Bożek

**Affiliations:** Clinical Department of Internal Disease, Dermatology and Allergology, Medical University of Silesia, 40-055 Katowice, Poland; mrozm@onet.pl (M.M.); szymuska44@gmail.com (S.M.); martynamiodonska298@gmail.com (M.M.); dziolkowska@op.pl (D.Z.); ewahadas@interia.pl (E.H.)

**Keywords:** psoriasis, psoriatic arthritis, COVID-19, biological drugs

## Abstract

*Background and objectives:* Biological treatment is an important and effective therapy for psoriasis. During the COVID-19 pandemic, it remains unclear whether this type of therapy affects the course of SARS-CoV-2 infection. The aim of the study was to observe patients with psoriasis undergoing biological or other systemic treatment in relation to the impact of SARS-CoV-2 infection on the course of psoriasis and the COVID-19 disease itself. *Materials and methods:* A one-year observational study included 57 patients with diagnosed psoriasis who qualified for biological treatment and a group of 68 similar patients who were administered a different systemic treatment. Patients were analyzed monthly for psoriasis (including Psoriasis Area Severity Index (PASI) assessment) and constantly for SARS-CoV-2 infection (telephone contact). Cases of COVID-19 were confirmed by Polymerase Chain Reaction (PCR) at the study center. *Results*: SARS-CoV-2 infection was confirmed by a positive Real Time Polymerase Chain Reaction (RT-PCR) test in eight patients (14.0%) with psoriasis on biological therapy. None of the cases in this group required hospitalization for COVID-19. Similar data were obtained in the control group. Specifically, 11 (16%) patients were confirmed to be infected with SARS-CoV-2. These results were statistically comparable (*p* > 0.05). In the group of patients undergoing biological treatment, six (75%) of eight patients developed an exacerbation of psoriasis during SARS-CoV-2 infection, and similar results were noted in the control group, with eight (72%) patients experiencing an exacerbation of psoriasis. *Conclusions*: Patients with psoriasis who were administered biological treatment or other systemic therapy may experience a mild course of SARS-CoV-2 infection but might also experience a temporary exacerbation of skin lesions.

## 1. Introduction

Psoriasis is a chronic, immune-mediated inflammatory skin disease. It is estimated that approximately 5–30% of patients with psoriasis vulgaris suffer from psoriatic arthritis (PsA) [1]. Psoriasis treatment includes the use of external preparations and systemic medications [1]. The use of biological drugs represents an important breakthrough in the treatment of psoriasis. These drugs inhibit effector cytokines, such as TNF-alpha, IL-12, IL-17, and IL-23, that play a key role in the pathomechanism of psoriasis [2].

Coronavirus disease (COVID-19) is caused by severe acute respiratory syndrome coronavirus 2 (SARS-CoV-2). The first cases were reported in Wuhan, China, in December 2019 [3]. This new virus has spread rapidly to many countries, and the ongoing SARS-CoV-2 pandemic presents us with new diagnostic and therapeutic challenges. Patients with severe psoriasis require the use of biologics to control disease severity, but it is unclear whether the achieved immunosuppression increases the risk of SARS-CoV-2 infection [4]. The aim of this study was to monitor patients with severe psoriasis who were administered biological therapy during the COVID-19 pandemic. Two key questions of this study were as follows: could the use of biological drugs have an impact on the course of COVID-19 infection? Could the use of biological drugs prevent symptomatic/asymptomatic SARS-CoV-2 infection?

## 2. Materials and Methods

This was a single-center, observational study. A group of 57 patients diagnosed with psoriasis and receiving biological treatment and a 68-person control group of similar patients with psoriasis not receiving biological treatment were observed from May 2020 to May 2021 in a clinical dermatological center.

The inclusion criteria for the study group were as follows: age > 18 years; diagnosis of severe psoriasis, including articular psoriasis; and patient qualifies for biological treatment. The diagnosis was based on a dermatological assessment and Psoriasis Area Severity Index (PASI) scales. Biological treatment had to be administered for a minimum of 1 year with any registered and commercially available drug for this type of treatment, namely secukinumab, adalimumab, ustekinumab, ixekizumab, or risankizumab.

Inclusion criteria for biological treatment included the following: age > 18 years; suffer from severe disease (or moderate in the case of treatment with infliximab), as determined by an assessment of the severity of the psoriatic process based on a PASI (Psoriasis Area Severity Index) score greater than 18, and either a DLQI (Dermatology Life Quality Index) score greater than 10 or a BSA (Body Surface Area) score greater than 10; and the patient did not improve as a result of treatment with at least two conventional agents, including methotrexate, cyclosporine, retinoids, or the Psoralen Ultra- Violet A (PUVA) method, in the period preceding the assessment for qualification.

In addition, the use of topical medications, nonsteroidal anti-inflammatory drugs (NSAIDs), glucorticosteroids, cygnoline (ditranol, antraline), vitamin D derivatives (e.g., tacalcitol), retinoids, tar preparations, and salicylic acid was permitted. The inclusion criteria for the control group were as follows: age > 18 years; severe psoriasis; no biological treatment or other systemic immunosuppressive treatments (cyclosporine, methotrexate, retinoid, or PUVA). Additionally, the topical medications described above were allowed.

### 2.1. Observation Method

Patients were monitored monthly at the center with a dermatological examination and administration of a biological drug or conventional therapy (control group). Additionally, patients were assessed for SARS-CoV-2 infection after every phone contact and were asked about the following: symptomatic infection, exposure to asymptomatic carriers, and hospital stay due to COVID. During the study, attempts were made to evaluate the course of psoriasis indirectly and, whenever possible, during the patient’s direct visit to the clinic (after the end of quarantine). The use of other drugs for the treatment of SARS-CoV-2 infection was also assessed. Additionally, telephone visits were performed in the event of a sudden exacerbation of the disease or suspicion or diagnosis of COVID-19.

Only dermatologists, with the help of additional medical personnel, conducted the assessments.

### 2.2. RT-PCR Diagnostics

The SARS-CoV-2 Real Time Polymerase Chain Reaction (RT-PCR) test is a real-time reverse transcription of the polymerase chain reaction for the qualitative detection of viral nucleic acids in the upper and lower respiratory tracts. Samples were taken from individuals suspected of being infected with SARS-CoV-2. Medical staff obtained samples from patients by taking swabs from the throat and atria of the nose. Nucleic acids were extracted from the samples using a MagNA Pure 96 system (Roche, Basel, Switzerland). A real-time targeted RT-PCR assay of the RdRp/Hel SARS-CoV-2 gene was performed using a Quanti Nova Probe RT-PCR kit (QIAGEN, Hamburg, Germany).

The characteristics of the groups are presented in Table 1.

## 3. Results

**Figure 1:**
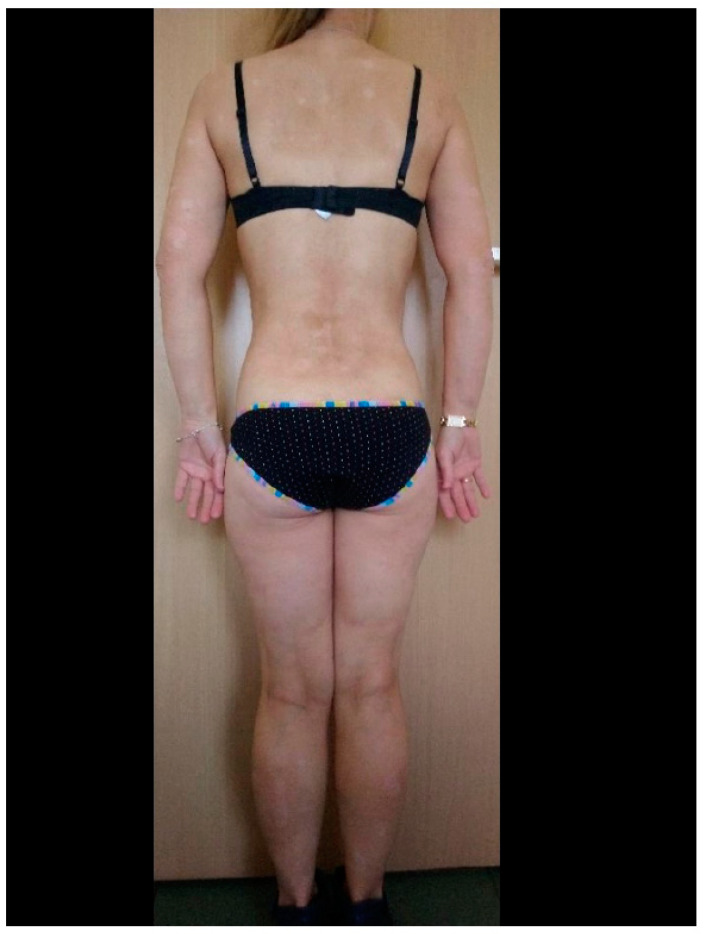
Patient before COVID-19 during biological therapy.

**Figure 2:**
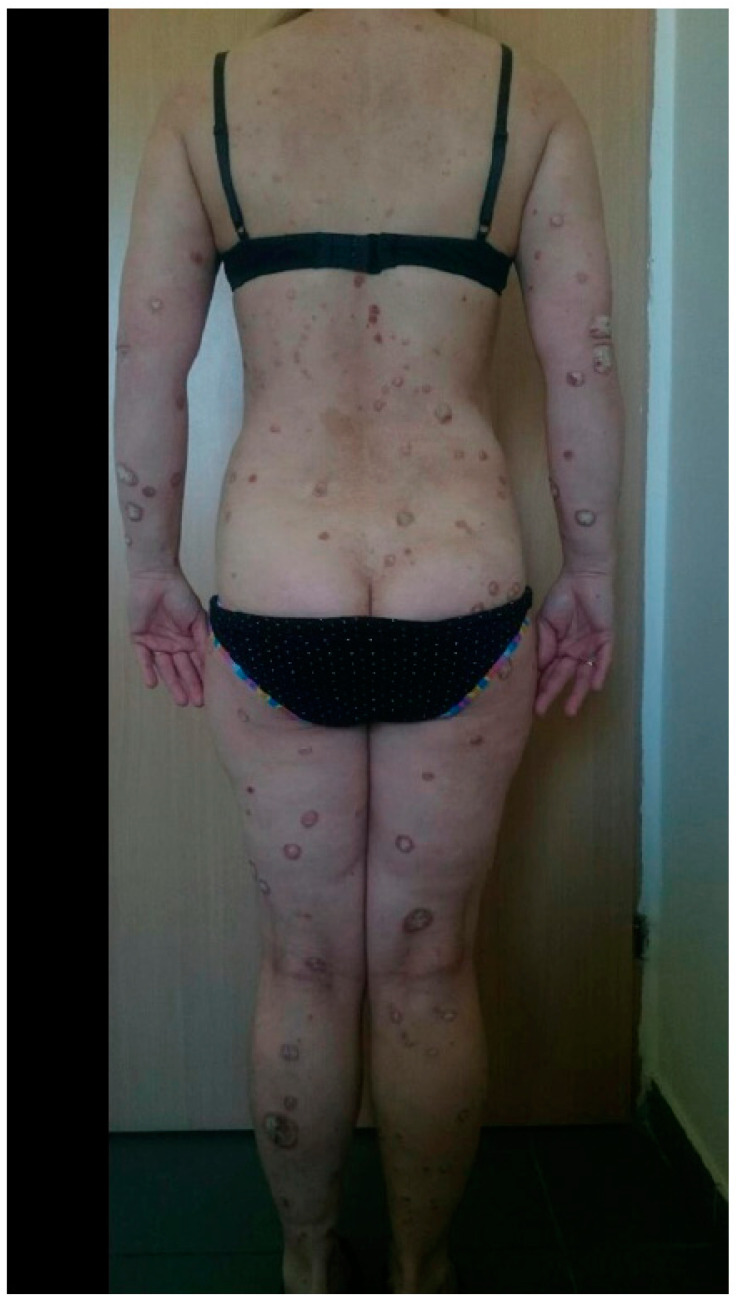
The same patient just after SARS-Co-V-2 infection.

In the group of patients under biological treatment, SARS-CoV-2 infection was confirmed by a positive RT-PCR test in eight patients (14.0%). All of these patients had mild clinical symptoms, including anosmia, cough, mild breathing difficulties, and fever. Hospitalization due to COVID-19 did not occur in this group. Similar data were obtained in the control group: 11 (16%) patients were confirmed to be infected with SARS-CoV-2. In this group, only mild disease was observed, and hospitalization was not required for any patient. These results were statistically comparable (*p* > 0.05). In the group of patients undergoing biological treatment, six (75%) of eight patients developed an exacerbation of psoriasis during SARS-CoV-2 infection (example of a patient before and immediately after SARS-CoV-2 infection: Figure 1 and Figure 2). A similar relationship was observed in the control group, in which 8 (72%) of 11 patients with SARS-CoV-2 infection experienced an exacerbation of psoriasis. Detailed data are presented in Table 2. In any described patients, psoriasis therapy was not suspended in the case of SARS-CoV-2 infection. In all patients with an exacerbation of psoriasis, the condition resolved within 4–6 weeks from the diagnosis of COVID-19 and did not require intensification of systemic treatment (local treatment was increased), with the exception of two patients on cyclosporin for whom the dose was temporarily increased (4–6 weeks) by 50%. There was no situation in which the patients themselves modified their psoriasis treatment.

## 4. Discussion

With the onset of the COVID-19 pandemic, there were alarming case reports of exacerbation of psoriasis after SARS-CoV-2 infection in patients not being treated with biological therapy [5,6,7].

The impact of SARS-CoV-2 infection on biologically treated patients with moderate to severe psoriasis is currently unclear. The primary immune response is satisfactory and leads to a decrease in viral load. Unfortunately, for reasons that remain unclear, the secondary immune response (the so-called “cytokine storm”) may be excessive and may be a factor leading to tissue integrity disorders, respiratory failure, or exacerbation of skin lesions. We cannot exclude the notion that biologics may reduce the production of pro-inflammatory cytokines, thereby reducing the negative impact of COVID-19 on the symptoms of psoriasis [5,6,7,8,9,10].

The presented work documents that all patients undergoing biological treatment had a mild course of COVID-19 and did not require hospitalization. Earlier studies also confirmed that the use of biological drugs by patients with psoriasis was associated with a much lower risk of hospitalization, although other factors, such as a patient’s age or comorbidities, should not be overlooked [11,12,13]. The current Group for Research and Assessment of Psoriasis and Psoriatic Arthritis (GRAPPA) guidelines recommend that biological treatments for psoriasis and psoriatic arthritis should not be discontinued or reduced during the pandemic, or when SARS-COV-2 virus infection is diagnosed. Research indicates that inappropriate treatment modification or discontinuation of treatment may worsen symptoms and reduce the response to re-treatment [14,15].

There are no conclusive data on the safety of initiating biological therapy in patients with psoriasis. In addition, a joint decision by a patient and their physician on the initiation of treatment is recommended, given that delayed treatment of psoriasis can seriously affect a patient’s physical and mental health [14,15,16].

The observed control group reacted in a similar manner to SARS-CoV-2 infection as patients on biological treatment. Specifically, no severe cases of COVID-19 and no cases requiring hospitalization were noted in either group. All patients were only symptomatically treated and did not require systemic steroids, heparin, or oxygen therapy. The patients only used antipyretic drugs.

None of our patients modified or suspended psoriasis treatment during SARS-Co-V-2 infection, and this excludes any additional impact on the course of psoriasis during COVID-19. This is different from other observations in which patients themselves decided to change or suspend such treatment [17]. It should be emphasized that, in the studied group of patients, a smaller than expected effect was observed in several patients after one year of biological treatment, which was often due to poor adherence (limitations related to the COVID-19 pandemic). It seems that completely uncontrolled psoriasis in individual patients could lead to instability during SARS-Co-V-19 infection.

It can be assumed that the transient exacerbations of psoriasis are associated with viral infection as a non-specific factor, which is similar to what is often observed with other infections in patients with psoriasis. However, due to the small study group, it was difficult to assess the impact of drugs on the course of SARS-CoV-2 infection. This is a significant limitation of this study. However, a similar observational study in patients with atopic dermatitis and immunosuppressive therapy also found no effect on the severity of COVID-19 [18]. The possible negative effects of this type of therapy should be emphasized. In the case of biological treatment, there are indications of an increased risk of infection in adult psoriasis patients treated with biological drugs. An analysis of data from 11,466 adults with psoriasis in the Psoriasis Longitudinal Assessment and Registry revealed an increased risk of serious infections with adalimumab and infliximab compared with and without non-biological systemic therapy. The rates of serious infections among patients treated with infliximab, adalimumab, etanercept, and ustekinumab were 2.49, 1.97, 1.47, and 0.83 per 100 patient-years, respectively [19]. No similar infections were recorded in the follow-up.

## 5. Conclusions

Patients with psoriasis who were administered biological treatment or other systemic therapy may experience a mild course of SARS-CoV-2 infection but might also experience a temporary exacerbation of skin lesions.

## Figures and Tables

**Table 1:** The characteristics of the groups.

	Study Group *n* = 57	Control Group *n* = 68
age, mean, SD (range)	50 ± 12.34 (22–74)	48 ± 15.21 (23–73)
sex	(%)	(%)
women	31.58 (18)	38.24 (26)
men	68.42 (39)	61.76 (42)
type of psoriasis according to ICD-10		
L.40.0 psoriasis vulgaris	47 (82)	54 (80)
L. 40.5 articular psoriasis	10 (18)	14 (15)
mean PASI before treatment	56 ± 11	49 ± 16
mean PASI after a year of treatment	8 ± 6	10 ± 6
type of systemic treatment		
secukinumab	29(51)	-
adalimumab	17 (30)	-
ustekinumab	8 (14)	-
ixekizumab	2 (4)	-
risankizumab	1 (2)	-
Cyclosporine ^a^		10 (15)
Methotrexate ^b^		21(31)
Retinoids ^c^		3(4)
PUVA ^d^		20 (29)
retinoids +PUVA		10 (15)
only topical treatment		4 (6)

Legend: mean daily dose of ^a^ 200 mg, ^b^ 12.5 mg, ^c^ acitrecin 30 mg, ^d^ UVA with the use 8-methoxypsoralen at a dose of 0.8 mg/kg or 5-methoxypsoralen at a dose of 1.2–1.4 mg/kg, SD: standard deviation, PASI: Psoriasis Area Severity Index, PUVA: Psoralen Ultra- Violet A.

**Table 2:** Characteristics of psoriasis patients who developed SARS-CoV-2 infection.

N S/C	Sex	Age	Type of Psoriasis According to ICD-10	Drug Name	Exacerbation of Psoriasis PASI Score before vs. after	Concomitant Diseases	Time of Exacerbation (days)
1S	M	72	L40.0	adalimumab	No 32–28	Arterial hypertension	-
2S	M	60	L40.0	secukinumab	No 29–31	-	-
3S	W	50	L40.0	ustekinumab	Yes 4–64	Obesity	12
4S	M	44	L40.0	ustekinumab	Yes 23–51	-	24
5S	W	43	L40.5	adalimumab	Yes 18–33	-	28
6S	M	50	L40.0	risankizumab	Yes 4–31	Arterial hypertension	28
7S	W	52	L40.0	ustekinumab	Yes 8–44	-	21
8S	W	52	L40.0	adalimumab	Yes 15–25	-	36
1C	M	51	L40.5	cyclosporin	No 17–15	-	-
2C	M	42	L40.5	PUVA	Yes 32–46	Arterial hypertension	24
3C	M	45	L40.0	cyclosporin	Yes 28–39	-	38
4C	W	62	L40.0	PUVA	Yes 12–54	-	42
5C	W	55	L40.0	cyclosporin	No 19–42	-	-
6C	M	54	L40.5	cyclosporin	Yes 33–63	Arterial hypertension	32
7C	M	46	L40.0	methotrexate	No 8–7	-	-
8C	W	69	L40.0	methotrexate	Yes 5–26	Diabetes Arterial hypertension	25
9C	M	38	L40.0	cyclosporin	Yes 9–52	-	60
10C	M	44	L40.0	PUVA	Yes 12–33	-	24
11C	W	49	L40.0	methotrexate	Yes 25–38	-	32

Legend: S—study group, C—control group, M: man, W: woman.

## Data Availability

The data presented in this study are available on request from the corresponding author.
